# Expression of the Major and Pro-Oncogenic H3K9 Lysine Methyltransferase SETDB1 in Non-Small Cell Lung Cancer

**DOI:** 10.3390/cancers11081134

**Published:** 2019-08-08

**Authors:** Paola Cruz-Tapias, Vlada Zakharova, Oscar M. Perez-Fernandez, William Mantilla, Sandra Ramírez-Clavijo, Slimane Ait-Si-Ali

**Affiliations:** 1 Epigenetics and Cell Fate (EDC), Centre National de la Recherche Scientifique (CNRS), Université de Paris, Université Paris Diderot, F-75013 Paris, France; 2 Grupo de investigación Ciencias Básicas Médicas, Faculty of Natural Sciences and Mathematics, Universidad del Rosario, Bogotá 111221, Colombia; 3 School of Medicine and Health Sciences, Universidad del Rosario, Bogotá 111221, Colombia; 4 Doctoral Program in Biomedical and Biological Sciences, Universidad del Rosario, Bogotá 111221, Colombia; 5 Department of Cardiology, Fundación Cardioinfantil - Instituto de Cardiología, Bogotá 110131, Colombia; 6 Department of Hematology-oncology. Fundación Cardioinfantil - Instituto de Cardiología, Bogotá 110131, Colombia

**Keywords:** SETDB1/KMT1E, lysine methyltransferase, non-small cell lung cancer, meta-analysis

## Abstract

SETDB1 is a key histone lysine methyltransferase involved in gene silencing. The *SETDB1* gene is amplified in human lung cancer, where the protein plays a driver role. Here, we investigated the clinical significance of *SETDB1* expression in the two major forms of human non-small cell lung carcinoma (NSCLC), i.e., adenocarcinoma (ADC) and squamous cell carcinoma (SCC), by combining a meta-analysis of transcriptomic datasets and a systematic review of the literature. A total of 1140 NSCLC patients and 952 controls were included in the association analyses. Our data revealed higher levels of *SETDB1* mRNA in ADC (standardized mean difference, SMD: 0.88; 95% confidence interval, CI: 0.73–1.02; *p* < 0.001) and SCC (SMD: 0.40; 95% CI: 0.13–0.66; *p* = 0.003) compared to non-cancerous tissues. For clinicopathological analyses, 2533 ADC and 903 SCC patients were included. Interestingly, *SETDB1* mRNA level was increased in NSCLC patients who were current smokers compared to non-smokers (SMD: 0.26; 95% CI: 0.08–0.44; *p* = 0.004), and when comparing former smokers and non-smokers (*p* = 0.009). Furthermore, the area under the curve (AUC) given by the summary receiver operator characteristic curve (sROC) was 0.774 (Q = 0.713). Together, our findings suggest a strong foundation for further research to evaluate SETDB1 as a diagnostic biomarker and/or its potential use as a therapeutic target in NSCLC.

## 1. Introduction

Lung cancer causes more than 1.6 million deaths per year worldwide, despite current progress in treatment [1]. The two major lung cancer types are non-small cell lung cancer (NSCLC) and small cell lung cancer (SCLC). The lung cancer mortality rate is driven by the high possibility of metastasis and problems in early diagnosis [2,3]. Lung cancer is a complex disease, which involves both genetic and epigenetic alterations (reviewed in [4]).

The combined use of next-generation high-throughput sequencing (NGS) and ultra-sensitive mass spectrometry technologies has substantially improved our understanding of molecular epigenetic mechanisms, such as histone post-translational modifications (PTMs) and transcriptional regulation in normal and pathological conditions, especially in cancer. Histone PTMs are one of the most important mechanisms of epigenetic regulation of gene expression and chromatin organization. As such, histone lysine methylation is a key regulatory mechanism of chromatin organization. Histone lysine methylation status is regulated by histone lysine methyltransferases (KMTs) and lysine demethylases (KDMs). A large number of studies have substantiated the link between aberrant histone lysine methylation and malignancy, and the roles of KMTs in cancer metastasis [5]. In particular, the methylation of histone 3 lysine 9 (H3K9), which is directly involved in heterochromatin formation and both gene repression and silencing [6]. The main H3K9 KMTs, namely, G9A, GLP, SUV39H and SETDB1, are deregulated in many cancers, and variations in the global or local patterns of H3K9 methylations are found in tumor cells. For instance, abnormal H3K9 methylations have been associated with poor survival and higher risk of relapse [7]. A loss of H3K9 dimethylation (H3K9me2) has been found in prostate, lung and kidney cancer patients [8,9], and H3K9me3 is a diagnostic marker of metastasis in lung cancer patients [10]. In addition, mutations in KMT genes or abnormal expression of KMTs are found in tumors [11].

SETDB1 (also named KMT1E) is a major H3K9 KMT known to be required for mammalian development as it regulates pluripotency in the early embryo [12], stem cell potential and terminal differentiation in many progenitor cell types [13,14]. SETDB1 is central in embryonic stem cell (ESC) pluripotency and self-renewal [15,16,17] and in many adult stem cells. Interestingly, tumors consist of heterogeneous cell populations and a subset of cells, so called cancer stem cells (CSCs), which express pluripotency markers and have the ability for self-renewal, such as ESCs. CSCs have been proposed as an origin for certain types of tumors, and the expression of pluripotency markers might hereby play a role [18]. Thus, SETDB1 key roles in stemness regulation may provide a link between ESCs and CSCs biology. We participated in a study showing that the human *SETDB1* gene is amplified in melanoma, in which SETDB1 accelerates tumorigenesis [19].

Furthermore, SETDB1 is overexpressed in lung cancer [20] and silences certain genes by direct interaction with the DNA methyltransferase DNMT3A [21], and both are implicated in epithelial-to-mesenchymal transition (EMT) and metastasis [22]. Most interestingly, an amplification of the *SETDB1* gene was also described in lung cancer, in which SETDB1 is considered as a pro-oncogene able to increase tumor invasion [23]. The *SETDB1* gene was found to be amplified in lung cancer cell lines and primary tumors [23]. The same study showed that the *SETDB1* gene is amplified several times in human NSCLC and SCLC cell lines and in primary lung tumors. The authors observed an increase in *SETDB1* copy number. Importantly, SETDB1 protein overexpression was associated with elevated cell growth rates and the invasive potential of cancer cells in nude mouse models [23]. High levels of *SETDB1* expression are also associated with poor prognosis in terms of overall survival of patients [24]. SETDB1 hyperactivation affects various signaling pathways such as WNT, MAPK, Toll-like receptors (TLRs), focal adhesion, and JAK-STAT pathways in lung cancer cells [24].

Here, we tested the clinical significance of *SETDB1* expression in NSCLC, based on the analysis of large-scale transcriptomic datasets. To this end, we conducted different meta-analyses using 45 microarray datasets from the Gene Expression Omnibus (GEO) database. Furthermore, summary receiver operator characteristic curve (sROC) analysis was used to determine the discriminative yield of *SETDB1* expression in NSCLC. In parallel, a systematic review of the literature was conducted up to April 2019 to provide information about the association of *SETDB1* expression and NSCLC. Our results showed higher levels of *SETDB1* mRNA in both ADC and SCC tissues compared to non-cancerous tissue controls. Interestingly, *SETDB1* mRNA level was increased in former or current smoker NSCLC patients compared to non-smokers.

Our findings suggest that *SETDB1* expression levels could be used as a diagnostic biomarker and/or potentially be used as a therapeutic target in NSCLC.

## 2. Results

### 2.1. Association Between SETDB1 Expression and NSCLC

**Figure 1:**
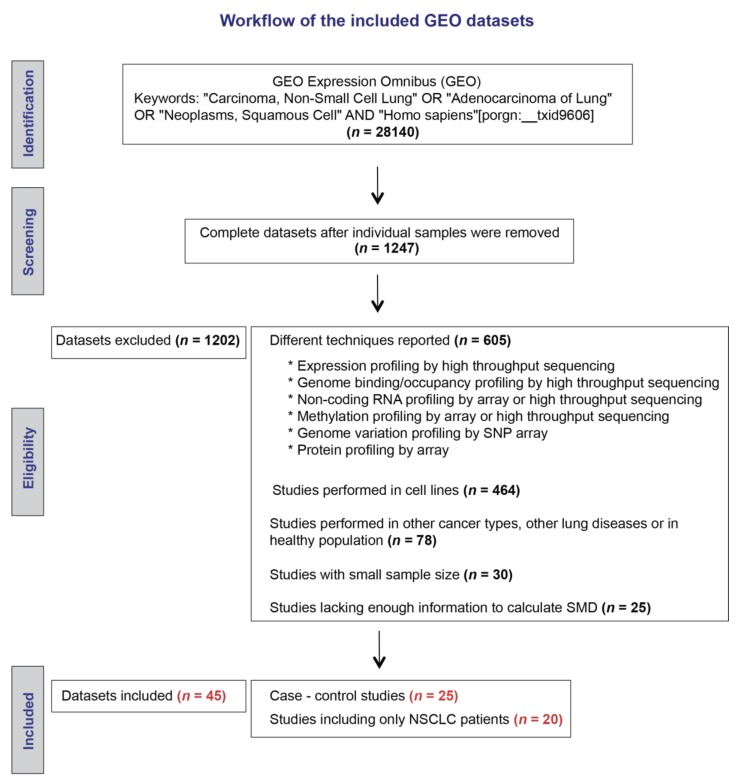
Workflow of the included Gene Expression Omnibus (GEO) datasets.

Our initial search strategy identified 1247 microarray datasets (Figure 1). After screening and eligibility assessment, we included a total of 20 datasets reporting expression levels of *SETDB1* mRNA in cancerous and adjacent non-cancerous specimens that were resected from NSCLC patients. In addition, a total of five datasets reporting expression levels of *SETDB1* in cancerous specimens from NSCLC patients and normal specimens from a healthy control group were included for meta-analysis. The main characteristics of the elected studies are described in Table 1.

**Figure 2:**
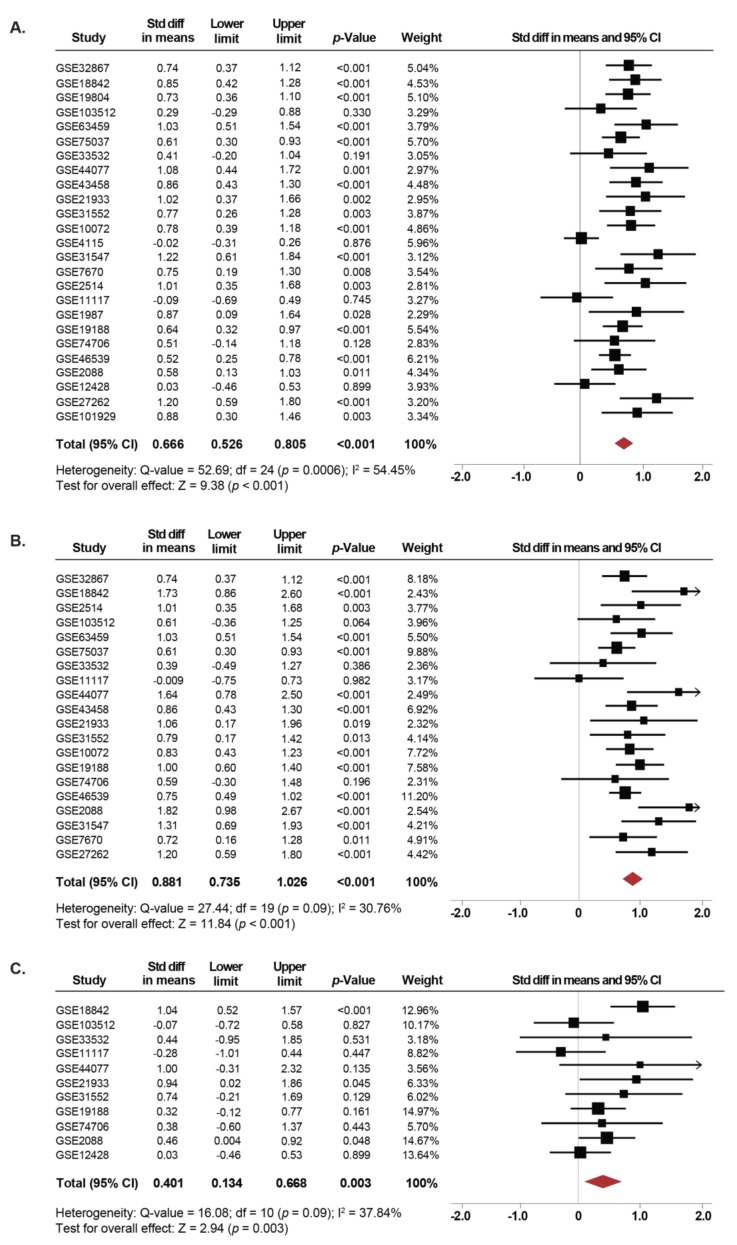
*SETDB1* mRNA expression is significantly increased in NSCLC patients. (**A**) Forest plot of standardized mean difference (SMD) comparing *SETDB1* mRNA levels in NSCLC patients and non-cancerous controls, including adjacent non-cancerous specimens that were resected from NSCLC patients or normal specimens from a healthy control. (**B**) and (**C**) Subgroup meta-analysis of the *SETDB1* mRNA levels between ADC patients (**B**) and SCC patients (**C**) compared to non-cancerous controls. Standardized mean differences for each dataset are represented by the squares, and the horizontal line crossing the square represents the 95% CI. The diamonds represent the estimated overall effect. The arrows indicate that the upper limit of the SMD is higher than 2.

Overall, 1140 NSCLC cases and 952 controls were analyzed. The expression of *SETDB1* was significantly increased in NSCLC tissues compared to normal lung tissues (SMD: 0.66; 95% CI: 0.52–0.80; *p* < 0.001) with moderate heterogeneity (*I^2^* = 54.4%; *p* < 0.001) (Figure 2A). Independent analyses of the association between *SETDB1* mRNA levels and NSCLC for each GEO dataset are presented in the Supplementary Figure S1. There was no evidence of publication bias based on the funnel plot and Egger’s test, as seen in Supplementary Figure S2A.

Subgroup analyses were performed for the most common subtypes of NSCLC, namely, adenocarcinoma (ADC) and squamous cell carcinoma (SCC). Interestingly, the expression of *SETDB1* was significantly increased in both, tissues from patients with ADC (SMD: 0.88; 95% CI: 0.73–1.02; *p* < 0.001) as well as SCC (SMD: 0.40; 95% CI: 0.13–0.66; *p* = 0.003) compared to non-cancerous lung tissues (Figure 2B,C). The heterogeneity was moderate for ADC (*I^2^* = 30.7%; *p* = 0.09) and SCC (*I^2^* = 37.8%; *p* = 0.09). There was no evidence of publication bias based on the funnel plot and Egger’s test (Figure S2B,C).

Thus, a global increase in *SETDB1* mRNA level seems to be a hallmark of NSCLC, both of ADC and SCC subtypes.

### 2.2. Expression of SETDB1 Is Increased in Current and Former Smokers Compared to NSCLC Non-Smoker Patients

**Figure 3:**
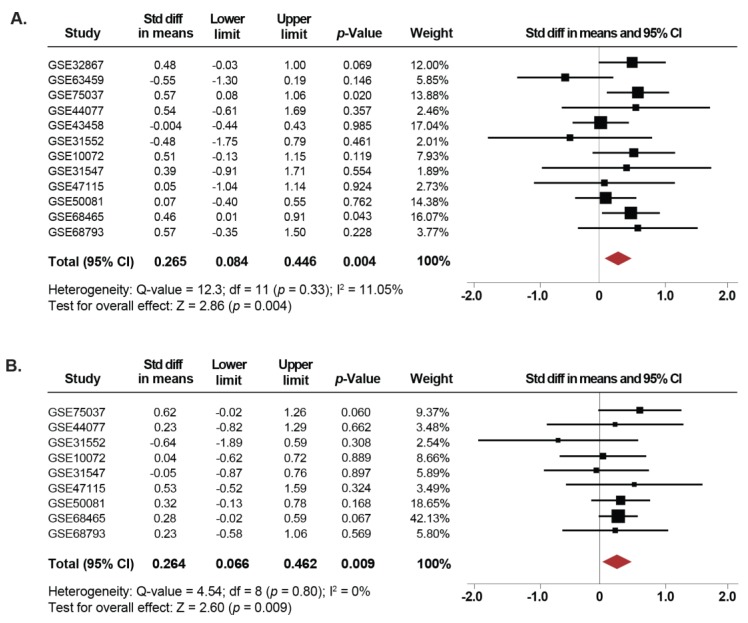
Upregulation of *SETDB1* mRNA is correlated with smoking history. (**A**) Forest plot of overall analysis of *SETDB1* mRNA expressions between NSCLC patients reported as current smokers compared to non-smoker patients. (**B**) Forest plot of overall analysis of *SETDB1* mRNA expressions between NSCLC patients reported as former smokers compared to non-smoker patients. Standardized mean differences (SMD) for each dataset are represented by the squares, and the horizontal line crossing the square represents the 95% CI. The diamonds represent the estimated overall effect.

Since tobacco use is the leading cause of lung cancer [1], we next investigated whether the expression of *SETDB1* is associated with smoking in NSCLC. Thus, we compared the changes in *SETDB1* mRNA levels between current, former and non-smoker NSCLC patients. Twelve microarray datasets, including 297 current smoker, 547 former smoker and 220 non-smoker NSCLC patients were used in this meta-analysis. Our results showed that *SETDB1* mRNA levels were increased in tissues from NSCLC patients who were current smokers compared to non-smokers (SMD: 0.26; 95% CI: 0.08–0.44; *p* = 0.004), with low heterogeneity (*I^2^* = 11%; *p* = 0.33) (Figure 3A). Furthermore, the same association was found when comparing NSCLC tissues from former smokers and non-smokers (SMD: 0.26; 95% CI: 0.06–0.46; *p* = 0.009), with low heterogeneity (*I^2^* =0%; *p* = 0.80) (Figure 3B). In addition, for 12 datasets reporting only whether the patients were smokers or non-smokers, an independent analysis was performed, showing that *SETDB1* mRNA levels were higher in smokers (SMD: 0.19; 95% CI: 0.05–0.33; *p* = 0.006), with low heterogeneity (*I^2^* = 0%; *p* = 0.503) (Figure S3A). There was no evidence of publication bias based on the funnel plot and Egger’s test (Figure S2D–F).

Furthermore, to evaluate whether the association of *SETDB1* mRNA levels with smoking status correlated with a cancer subtype, subgroup analyses were performed for ADC patients. Herein, *SETDB1* mRNA expression was higher in tissues from patients with ADC who were current smokers compared to non-smokers (SMD: 0.35; 95% CI: 0.03–0.67; *p* = 0.027), with moderate heterogeneity (*I^2^* = 44.3%; *p* = 0.072). Also, a trend was observed, approaching statistical significance, showing an increase in expression levels of *SETDB1* in ADC tissues from former smokers (SMD: 0.25; 95% CI: −0,005–0.52; *p* = 0.055), with low heterogeneity (*I^2^* = 21.2%; *p* = 0.26). Subgroup analyses for SCC patients were not possible because of an insufficient number of non-smoker patients. There was no evidence of publication bias based on the funnel plot and Egger’s test.

Finally, there were no clinical or pathological characteristics (gender, age and clinical stage) associated with the smoking status of the NSCLC patients and the increase in *SETDB1* expression levels (Table 2).

Together, these data indicate that higher levels of *SETDB1* mRNA correlate with the patient’s smoking history status.

### 2.3. Association Between SETDB1 Levels and Pathological Characteristics of the NSCLC Samples

Overall, 34 microarray datasets containing information about pathological characteristics were analyzed (Table 1 and Table S1). There was no statistical evidence that pathological stages or tumor-node-metastasis (TNM) stages in ADC and SCC have an association with the expression levels of *SETDB1* (Table 2). However, as shown in Supplementary Figure S3B, a possible trend toward significance showing an association between *SETDB1* expression and NSCLC patients carrying mutations for the *TP53* gene was observed (SMD: 0.15; 95% CI: −0.004–0.31; *p* = 0.052), with low heterogeneity (*I^2^* = 0%; *p* = 0.60). There was no evidence of publication bias based on the funnel plot and Egger’s test (Figure S2G). No associations were found between *SETDB1* expression and NSCLC patients carrying mutations for neither the *Epidermal Growth Factor Receptor* (*EGFR*) nor the *KRAS* oncogenes (Table 2).

Collectively, these findings suggest that high levels of *SETDB1* mRNA are not dependent on an NSCLC patient’s gender or age and are maintained at every clinical stage during the carcinogenic process.

### 2.4. Diagnostic Value of SETDB1 in NSCLC, Based on Meta-Analysis

**Figure 4:**
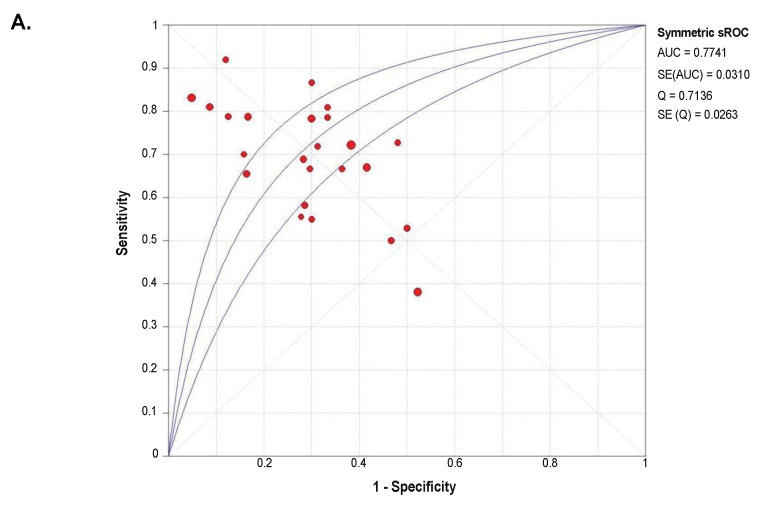

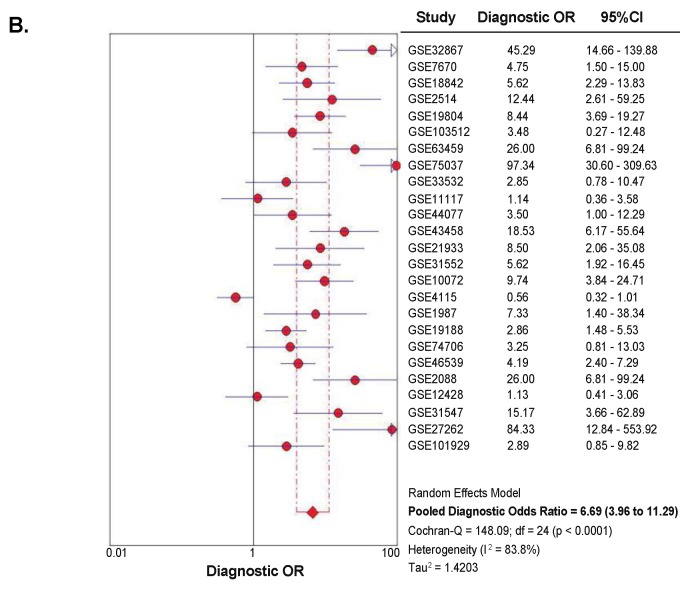
Summary receiver operator characteristic curve (sROC) and diagnostic odds ratio (OR), showing a moderate discriminative yield of *SETDB1* mRNA expression for NSCLC. (**A**) Symmetrical summary receiver operator characteristic curve (sROC) based on 1140 NSCLC tissues and 952 non-cancerous tissues (25 GEO datasets). The size of the circle symbolizes the sample size of each study included in the meta-analysis. (**B**) Forest plot for diagnostic odds ratios in NSCLC diagnosis. The circles represent odds ratios for each dataset. The diamond represents the estimated overall effect, based on the meta-analysis random-effect method.

A summary receiver operator characteristic curve (sROC) analysis was carried out to determine the discriminative yield of *SETDB1* mRNA level in NSCLC. A total of 1140 patients from 25 GEO microarray datasets were included in this analysis. Detailed information and independent ROC curves of each record are presented in Supplementary Figure S4. The overall combined area under the sROC curve was 0.774 (Standard Error, SE = 0.031), and the diagnostic odds ratio was 6.69 (95% CI: 3.96–11.29), with high heterogeneity (*I^2^* = 83.8%; *p* < 0.0001) (Figure 4). The combined sensitivity and specificity were 0.69 (95% CI: 0.66–0.72) and 0.71 (95% CI: 0.68–0.74), respectively (Figure S5**)**, with a pooled LR+ of 2.55 (95% CI: 1.95–3.35) and a pooled LR- of 0.41 (95% CI: 0.32–0.53).

Altogether, our analyses revealed that *SETDB1* expression yielded a moderate prediction value for NSCLC.

### 2.5. Literature Review on SETDB1 in NSCLC

A total of 13 records were retrieved from the databases Pubmed, EBI-EMBL, Web of Science, Embase, Bibliovie and Cochrane Library (Figure S6). The main results are recapitulated in Table 3 and Supplementary Table S2. In summary, the studies used more than 20 lung cancer cell lines, primary tissues and xenograft models.

Concerning the analysis of SETDB1 status in primary tissues, there are seven studies reporting the association of SETDB1 with the carcinogenic process in lung cancer (Table 3). Thus, the *SETDB1* gene is amplified in NSCLC tissues [23,24] and could be considered as a marker of a shorter survival period in ADC patients [49]. Moreover, *SETDB1* mRNA levels were higher in tissues from NSCLC patients compared to non-tumor tissues [24,50,51], and this increase was associated with advanced grade NSCLC tumors [24], shorter overall survival in NSCLC patients [50] and shorter disease-free survival in NSCLC patients in stage I [51]. Interestingly, the amplification of the *SETDB1* gene was correlated with high *SETDB1* mRNA levels [23,24] and protein overexpression in NSCLC tissues [23]. Finally, SETDB1 protein levels were higher in tissues from lung cancer patients compared to non-tumor tissues [24,50,52]. Sun et al. reported a possible trend correlating *SETDB1* expression with an advanced pathological state [24]. However, Wu et al. observed a higher expression of *SETDB1* during the early stages of lung cancer [52].

In addition, ten studies reported the pro-oncogenic role of SETDB1 in lung carcinogenesis, based on cell lines and xenograft models (Table S2**)**. In general, SETDB1 is associated with the regulation of cell proliferation, cellular invasion [20,23,24,52] and apoptosis [53]. Moreover, SETDB1 participates during the oncogenic process by the activation of different pathways, including WNT [24] and AKT, [50] or by the regulation of miRNAs, such as the miR-29 family [54]. Finally, three studies reported results highlighting the possible therapeutic targeting of SETDB1 during lung cancer treatment [55,56,57].

## 3. Discussion

Lysine methylation is a key post-translational modification that regulates gene expression at different levels, ranging from transcriptional to post-transcriptional and translational. For instance, lysine methylation affects the stability, localization and activity of proteins, such as proteins involved in cell signaling pathways and in the transcriptional and post-transcriptional regulation of gene expression; TP53, NF-κB, YAP and STAT3 are some examples of important methylated proteins [58,59,60,61]. Therefore, lysine methylation and its regulators have a key impact on normal cell fate and its deregulation in disease, such as in cancer.

Histone lysine methylation plays important roles in lung cancer development [10]. Dynamic histone lysine methylation status is regulated by the interplay among histone methyltransferases (KMTs) or demethylases (KDMs). The genes coding for these enzymes may be subject to mutations, chromosomal deletions or amplifications, and these factors change the overall histone methylation/demethylation balance. For example, recent publications reported that *SETDB1* is subject to gene amplification-associated activation in lung tumorigenesis [23]. SETDB1 might impact the cancer phenotype by acting on different substrates. Indeed, in addition to its best-known target, namely, H3K9, it is also known that SETDB1 methylates many other non-histone substrates with high relevance to lung cancer. These include the tumor suppressor TP53 and the kinase AKT [50,62]. Thus, SETDB1 overexpression in lung cancer cells could be crucial at different molecular levels, not only at the chromatin level.

Here, we asked whether *SETDB1* overexpression was related to the clinical features of lung cancer patients with two major types of NSCLC, namely, adenocarcinoma and squamous cell carcinoma.

We analyzed 25 published gene transcriptomic datasets and found that *SETDB1* mRNA level was significantly increased in NSCLC tissues compared to normal lung tissues. In many cases, the copy number gain or amplification of the *SETDB1* gene locus in primary tumors was accompanied with elevated *SETDB1* mRNA and protein levels [23,24,50,51,54]. *SETDB1* was also found to be amplified and/or upregulated in several NSCLC cell lines (NCI-H1437, NCI-H1395, A549, Calu-1, SK-MES-1, SK-LU-1, SW-900, and PC14) [20,23,54].

Our subgroup analyses for ADC and SCC showed higher *SETDB1* mRNA levels in ADC as compared to SCC, while *SETDB1* expression in both cancer subtypes was still significantly higher than in normal lung tissues. This is consistent with lower levels of *SETDB1* amplification in SCC compared to ADC [49].

We observed no statistically significant correlation between the clinical stage of ADC or SCC and *SETDB1* expression. Previously published studies differ on this issue. Indeed, Inoue et al. reported that *SETDB1* amplification in ADC was associated with an advanced cancer stage [49]. In contrast, Lafuente-Sanchis et al. showed that high *SETDB1* expression in NSCLC was observed at the earliest cancer stages [51]. In all cases, amplification and a high level of expression of *SETDB1* were associated with a shorter disease-free survival [49,50,51]. These discrepancies may be due to both the different selection criteria of the cases but also to a different number of patients included in these studies.

Several studies observed overexpression of *SETDB1* in other types of tumors, like hepatocellular carcinoma and melanoma, which was associated with a poor prognosis [19,63,64]. Importantly, the silencing of SETDB1 was shown to inhibit cell proliferation, cell invasion, tumor growth and metastasis in different types of cancer [65,66]. In vitro and in vivo experiments showed that *SETDB1* overexpression was associated with elevated cell growth rates and invasive potential of cancer cells in nude mouse models [20,23,50,55]. SETDB1 hyperactivation affects various signaling pathways, such as the WNT, MAPK, Toll-like receptors (TLRs), focal adhesion, and JAK-STAT pathways in lung cancer cells [24]. In particular, the WNT signaling pathway helps maintain cancer stem cells and correlates with an increased tumor growth and initial potential [67]. The major (canonical) WNT pathway signaling occurs through β-catenin [68]; abnormal expression of β-catenin is linked to the development of particular types of breast, colorectal, prostate and lung cancers [69]. Wang et al. demonstrated that SETDB1-mediated AKT methylation correlates with AKT hyperactivation in NSCLC, promotes tumor development and predicts poor outcome [50]. Chen et al. showed that SETDB1 negatively regulated the expression of *TP53* [54]. Indeed, Lafuente-Sanchis et al., with multivariate analysis, confirmed the independent prognostic value of SETDB1 for patients with the early stage of NSCLC [51].

As many other oncogenes, in certain conditions SETDB1 can participate in tumor suppression: the expression of *SETDB1* was significantly decreased in highly metastatic sublines of the CL1 lung cancer cell line (adenocarcinoma) [52], but at the same time *SETDB1* mRNA was high in the primary tumor samples in the early stages of NSCLC compared to the advanced stages. Accordingly, Wu et al. reported not only a pro-oncogenic role of SETDB1, but also an anti-oncogenic role in different stages of lung carcinogenesis, which is probably related to the cellular model chosen [52]. Thus, SETDB1 could play different roles in lung tumorigenesis. A strong correlation exists between high *SETDB1* expression and the earliest stage of NSCLC, supporting the role of the gene at least in the first step of lung tumorigenesis. At later stages, SETDB1 becomes dispensable for tumor progression and its expression diminishes, though it remains high compared to normal lung epithelial cells. This behavior is found with many oncogenes [70].

Our findings open up the possibility to use *SETDB1* expression level as a marker for early detection of patients at early stages of NSCLC and as a potential drug target in these patients.

## 4. Materials and Methods

### 4.1. Search Strategy for Microarray Databases in the Gene Expression Omnibus (GEO) Repository

Available microarray datasets related to NSCLC were downloaded from the GEO repository (https://www.ncbi.nlm.nih.gov/gds). The final date for inclusion was April 2019. The search strategy included the terms (“Carcinoma, Non-Small-Cell Lung” [Mesh]) AND (“Homo sapiens” [porgn:_txid9606]).

The inclusion criteria were the following: (1) enrolled data must be obtained from humans; (2) microarray datasets with information about *SETDB1* expression; (3) the sample type is not cell lines; (4) sufficient information to calculate the standardized mean difference (SMD); (5) for association analyses between *SETDB1* expression and NSCLC, two types of studies are included: (i) paired cancerous and adjacent non-cancerous tissues resected from NSCLC patients, (ii) cancerous specimens from NSCLC patients and normal specimens from a healthy control group. Importantly, the sample size must contain at least a ratio of 4:1 for cases and controls; (6) for clinical and pathological analyses, patients who had adenocarcinoma or squamous cell carcinoma with clinical information.

### 4.2. Data Extraction

Based on the inclusion criteria, the following detailed parameters were extracted: GEO accession number, PubMed identifier (PMID), sample type, cancer type (NSCLC, ADC or SCC), sample size, gender, age, cancer stage, smoking history and expression values of *SETDB1*, by using the tool GEO2R from the National Center for Biotechnology Information (NCBI).

### 4.3. Statistical Analysis

For each GEO dataset, the association between *SETDB1* expression and NSCLC was assessed by a Student’s t-test or a Mann–Whitney unpaired test based on normality distribution. Furthermore, to generate individual receiver operator characteristic (ROC) curves, the true positive (TP), false positive (FP), false negative (FN), and true negative (TN) values were estimated. All aforementioned analyses were performed using the Statistical Package for Social Sciences (SPSS Version 25, Chicago, IL, USA).

For meta-analysis, standardized mean difference (SMD) with 95% confidence interval (95% CI) was used as a summary statistic, considering the fact that all studies measured the same outcome but at different scales. Heterogeneity was calculated by means of Cochran’s (Q) and Higgins’s (*I^2^*) tests. The *I^2^* test was expressed as a ratio ranging from 0% to 100%. If *I^2^* > 30% and *p*-value < 0.05, the random-effects model was selected. Otherwise, the fixed-effects model was selected. A significant Q-statistic (*p* < 0.10) indicated heterogeneity across studies. To further evaluate the probable sources of heterogeneity, subgroup analyses were carried out. The presence of publication bias was graphically examined using funnel plots and Egger’s regression asymmetry tests. Data were analyzed using the Comprehensive Meta-Analysis version 2 program (Biostat, Englewood, NJ, USA 2004).

For diagnostic study, a summarized receiver operator characteristic curve (sROC) was constructed and the area under the sROC curve (AUC) was recorded, as well as the sensitivity and specificity. These analyses were performed using the MetaDiSc 1.4 software.

### 4.4. Search Strategy for Peer-Reviewed Journals

A systematic review of electronic databases (Pubmed, EBI-EMBL, Web of Science, Embase, Bibliovie and Cochrane Library) was done independently by two experts. The final date for inclusion was April 2019. The search included publications about the association of SETDB1 and NSCLC. The search strategy used MeSH terms (“Carcinoma, Non-Small-Cell Lung”[Mesh]) AND (“SETDB1 protein, human” [Supplementary Concept]). Only manuscripts published in a peer-reviewed journal as a full paper were included. Summaries or abstracts were not accepted.

## 5. Conclusions

Epigenetic mechanisms and regulators are often deregulated in human disease conditions. Thus, epigenetic mechanisms have gained paramount importance in biomedical research, since their reversibility provides new possibilities in therapeutic intervention. On the other hand, their expression status could have a prognostic and diagnostic value. Here, we tested the clinical significance of *SETDB1* mRNA level in lung cancer subtypes. Our overall pooled meta-analysis outcome revealed higher levels of *SETDB1* mRNA in NSCLC as compared to non-cancerous control tissues. Interestingly, *SETDB1* mRNA level was higher in tissues from NSCLC patients who were current or former smokers compared to non-smokers. Together, our findings suggest the possibility to use *SETDB1* mRNA level as a marker for NSCLC early detection and as a potential druggable target in these patients.

## Figures and Tables

**Table 1:** Overview of the datasets selected from GEO for case-control analyses.

GEO Dataset	Country	Year	ADC	SCC	NSCLC *	Controls	Sample Type in Patients	Sample Type in Controls	Platform	SETDB1 ID	Reference
GSE32867	Canada	2012	58			58	Cancer tissue	Adjacent non-cancerous tissues	Illumina Human WG-6 v3.0 Expression BeadChips	ILMN_1718207	[25]
GSE18842	Spain	2010	14	31		46	Cancer tissue	Adjacent non-cancerous tissues	Affymetrix Human Genome U133 Plus 2.0 Array	203155_at	[26]
GSE19804	Taiwan	2010			60	60	Cancer tissue	Adjacent non-cancerous tissues	Affymetrix GeneChip Human Genome U133 plus array	203155_at	[27]
GSE103512	Germany	2017	30	25		14	Cancer tissue	Adjacent non-cancerous tissues	Affymetrix HT-U133plus-2-PM microarrays	203155_PM_at	[28]
GSE63459	USA/Japan	2015	33			32	Cancer tissue	Adjacent non-cancerous tissues	Illumina HumanRef-8 v3 Expression Beadchip arrays	ILMN_1718207	[29]
GSE75037	USA	2016	83			83	Cancer tissue	Adjacent non-cancerous tissues	Illumina BeadChip array HumanWG-6 V3	ILMN_1718207	[30]
GSE33532	Germany	2014	10	4	6	20	Cancer tissue	Adjacent non-cancerous tissues	Affymetrix U133 Plus 2.0 arrays	203155_at	[31]
GSE44077	USA	2013	14	5	2	21	Cancer tissue	Adjacent non-cancerous tissues	Affymetrix Human Gene 1.0 ST Array [transcript (gene) version]	7905258	[32]
GSE43458	USA	2013	80			30	Cancer tissue	Adjacent non-cancerous tissues	Affymetrix Human Gene 1.0 ST Array [transcript (gene) version]	7905258	[33]
GSE21933	USA/Taiwan	2012	11	10		21	Cancer tissue	Adjacent non-cancerous tissues	Phalanx Human OneArray	PH_hs_0023897	[34]
GSE31552	USA	2014	21	9	2	32	Cancer tissue	Adjacent non-cancerous tissues	Affymetrix Human Gene 1.0 ST Array [transcript (gene) version]	7905258	[35]
GSE10072	Italy	2008	58			49	Cancer tissue	Healthy lung tissues	Affymetrix Human Genome U133A Array	203155_at	[36]
GSE4115	USA	2007				90	Cancer tissue	Healthy lung tissues	Affymetrix Human Genome U133A Array	203155_at	[37]
GSE31547	USA	2018	30			20	Cancer tissue	Adjacent non-cancerous tissues	Affymetrix Human Genome U133A Array	203155_at	Notpublished
GSE7670	Taiwan	2007	26			27	Cancer tissue	Adjacent non-cancerous tissues	Affymetrix Human Genome U133A Array	203155_at	[38]
GSE2514	USA	2005	20			19	Cancer tissue	Adjacent non-cancerous tissues	Affymetrix GeneChip microarray (HGU95Av2)	34189_at	[39]
GSE11117	Switzerland	2010	13	14		15	Cancer tissue	Chronic inflammatory lung disease tissues	Nova Chip microarrays (Novartis)	H200002955	[40]
GSE1987	Israel	2006			28	9	Cancer tissue	Healthy lung tissues	Affymetrix Human Genome U95A Array	34189_at	[41]
GSE19188	Netherlands	2010	45	27		65	Cancer tissue	Adjacent non-cancerous tissues	Affymetrix Human Genome U133 Plus 2.0 Array	203155_at	[42]
GSE74706	Germany	2016	10	8		18	Cancer tissue	Adjacent non-cancerous tissues	Agilent-026652 Whole Human Genome Microarray 4 × 44K v2	A_23_P126393	[43]
GSE46539	Taiwan	2015	115			115	Cancer tissue	Adjacent non-cancerous tissues	Illumina WG-DASL HumanRef8 v3 or HumanHT12 v4 BeadChip	ILMN_1718207	[44]
GSE2088	Japan	2011	9	48		30	Cancer tissue	Healthy lung tissues	CHUGAI 41K microarray	11758	[45]
GSE12428	Netherlands	2008		34		28	Cancer tissue	Adjacent non-cancerous tissues/Healthy lung tissues	Agilent-012391 Whole Human Genome Oligo Microarray G4112A	7231	[46]
GSE27262	Taiwan	2012	25			25	Cancer tissue	Adjacent non-cancerous tissues	Affymetrix Human Genome U133 Plus 2.0 Array	203155_at	[47]
GSE101929	USA	2017			25	25	Cancer tissue	Adjacent non-cancerous tissues	Affymetrix Human Genome U133 Plus 2.0 Array	203155_at	[48]

* Non-small cell lung carcinoma (NSCLC): Patients are not classified as adenocarcinoma (ADC) or squamous cell carcinoma (SCC).

**Table 2:** Analysis of the association between *SETDB1* mRNA expression and clinical and pathological characteristics.

Characteristics	Std Diff in Means	Lower Limit	Upper Limit	*p*-Value
**Clinical Characteristics**				
*Size and extent of the main tumor (T)*				
ADC T1 vs. T2/T3/T4	−0.03	−0.156	0.096	0.636
SCC T1 vs. T2/T3/T4	0.077	−0.192	0.346	0.574
*Spread to nearby lymph nodes (N)*				
ADC N0 vs. N1/N2/N3	0.028	−0.21	0.266	0.82
SCC N0 vs. N1/N2/N3	0.075	−0.137	0.287	0.489
*Stage of cancer*				
ADC 1 vs. 2/3/4	−0.023	−0.129	0.083	0.666
SCC 1 vs. 2/3/4	−0.038	−0.179	0.103	0.596
*Presence of mutations in genes*				
EGFR	0.088	−0.116	0.291	0.399
KRAS	0.114	−0.114	0.342	0.326
**Smoking Status**				
NSCLC—Age	0.007	−0.125	0.139	0.918
NSCLC—Gender	0.013	−0.131	0.158	0.857
ADC—Age	0.011	−0.106	0.128	0.853
ADC—Gender	0.012	−0.184	0.208	0.906
ADC—Stage 1 vs. 2/3/4	−0.106	−0.239	0.027	0.119
SCC—Age	0.07	−0.137	0.277	0.505
SC—Gender	−0.096	−0.339	0.148	0.441
SCC—Stage 1 vs. 2/3/4	0.068	−0.201	0.338	0.619

**Table 3:** Literature review on SETDB1 status in primary lung cancer tissues.

Study	Study Design and Subjects	Main Findings	Methods and Analysis	Conclusions
**Wu et al. 2014** [52]	Primary tumors of lung cancer patients at different clinical stages (*n* = 192) and adjacent normal tissues (*n* = 16).	SETDB1 protein levels were elevated in lung cancer tissues compared to non-tumor tissues. Possible association with early stages.	IHC, tissue microarray	SETDB1 is highly expressed in lung cancer.
**Rodriguez-Paredes et al. 2014** [23]	Primary ADC (*n* = 20), SCC (*n* = 20), SCLC (*n* = 19) tissues.	Amplification of the *SETDB1* gene correlates with elevated *SETDB1* transcripts and protein overexpression in tissues from patients with NSCLC and SCLC.	FISH, qPCR, IHC	*SETDB1* is amplified and highly expressed in NSCLC and SCLC.
**Sun et al. 2015** [24]	TCGA ADC dataset.	Amplification of *SETDB1* loci correlates with elevated *SETDB1* transcripts.	Bioinformatics	*SETDB1* is amplified and highly expressed in NSCLC.
	Eight microarrays from GEO and Expression Atlas databases. Primary NSCLC (*n* = 60) and their paired adjacent normal tissues (*n* = 60).	*SETDB1* mRNA levels were higher in NSCLC tissues compared to non-tumor tissues. Elevated expression of *SETDB1* correlates with advanced grade tumors.	Bioinformatics, RT-qPCR	
	Lung cancer tissues (*n* = 387) and normal bronchial epithelium cells (*n* = 106).	SETDB1 protein levels were elevated in lung cancer tissues compared to non-tumor tissues. Possible association with an advanced pathological stage.	IHC, tissue microarray	
**Inoue et al. 2015** [49]	Primary ADC (*n* = 164) and SCC (*n* = 99) tissues.	High-level amplification of the *SETDB1* gene in ADC tissues was associated with an advanced pathological stage.	FISH	*SETDB1* gene amplification is a marker of poor survival in ADC.
		Low-level amplification of the *SETDB1* gene was observed in SCC tissues.	FISH	
		*SETDB1* gene amplification was associated with shorter postoperative overall survival in ADC patients.	Kaplan-Meier analysis	
**Lafuente-Sanchis et al. 2015** [51]	Stage I primary NSCLC (*n* = 64) and adjacent normal tissues.	*SETDB1* mRNA was upregulated in primary NSCLC tissues. High *SETDB1* mRNA levels were associated with a shorter disease-free survival in stage I NSCLC.	RT-qPCR, Kaplan-Meier analysis	High mRNA levels of *SETDB1* as a prognostic marker of a shorter disease-free survival.
**Chen et al. 2018** [54]	Stage III and IV primary NSCLC tissues (*n* = 30) and paired adjacent normal tissues (*n* = 30).	*SETDB1* mRNA was upregulated in primary NSCLC tissues. *SETDB1* and *TP53* mRNA levels were negatively correlated.	RT-qPCR	*SETDB1* is highly expressed in NSCLC.
**Wang et al. 2019** [50]	Oncomine database: NSCLC tissues (*n* = 1926).	*SETDB1* mRNA was upregulated in NSCLC tissues. Higher expression of *SETDB1* mRNA was associated shorter overall survival in NSCLC patients.	Bioinformatics, Kaplan-Meier analysis	High mRNA levels of *SETDB1* as a prognostic marker of poor survival in NSCLC patients.
	Primary NSCLC tissues (*n* = 9) and paired adjacent normal tissues (*n* = 9).	SETDB1 was overexpressed in most paired NSCLC tumors compared to non-tumor tissues.	WB	SETDB1 is highly expressed in NSCLC.
	Primary NSCLC tissues (*n* = 156).	SETDB1 protein levels were elevated in NSCLC tissues (SETDB1 was detected in the nucleus and cytoplasm).	IHC	

Abbreviations: The Cancer Genome Atlas (TCGA), immunohistochemistry (IHC), fluorescence in situ hybridization (FISH), reverse transcription polymerase chain reaction (RT-qPCR), Western blot (WB).

## Supplementary Materials

The following are available online at https://www.mdpi.com/2072-6694/11/8/1134/s1, Figure S1: Box plots displaying the expression levels of *SETDB1* mRNA between NSCLC and non-tumor samples for each GEO dataset, Figure S2: Funnel plots for main meta-analyses performed., Figure S3: (A) Forest plot of the association of *SETDB1* mRNA level with NSCLC patients reported as smokers compared to non-smoker patients, (B) Forest plot of association between *SETDB1* mRNA level and NSCLC patients carrying mutations for TP53 gene, Figure S4: Individual ROC curves showing discriminative yield of *SETDB1* mRNA level for NSCLC for each GEO dataset, Figure S5: The combined sensitivity and specificity showing moderate discriminative yield of *SETDB1* mRNA expression for NSCLC, Figure S6: Workflow of the study selection for qualitative synthesis, Table S1: Overview of additional datasets selected from GEO for clinicopathological analyses, Table S2: Literature review about SETDB1 status in cell lines and xenograft models.
