# Supplementary material for: Expression of the Major and Pro-Oncogenic H3K9 Lysine Methyltransferase SETDB1 in Non-Small Cell Lung Cancer
Source: Cancers (Basel). 2019 Aug 8;11(8):1134. doi: 10.3390/cancers11081134 (PMC6721806; doi:10.3390/cancers11081134)
Supplement: Supplementary file 1 [file cancers-11-01134-s001.pdf]

Supplementary Materials: Expression of The Major and Pro-Oncogenic H3K9 Lysine Methyltransferase SETDB1 in Non-Small Cell Lung Cancer: A Systematic Review of the Literature and Meta–Analysis

Paola CRUZ-TAPIAS, Vlada ZAKHAROVA, Oscar M. PEREZ-FERNANDEZ, William MANTILLA, Sandra RAMÍREZ-CLAVIJO and Slimane AIT-SI-ALI

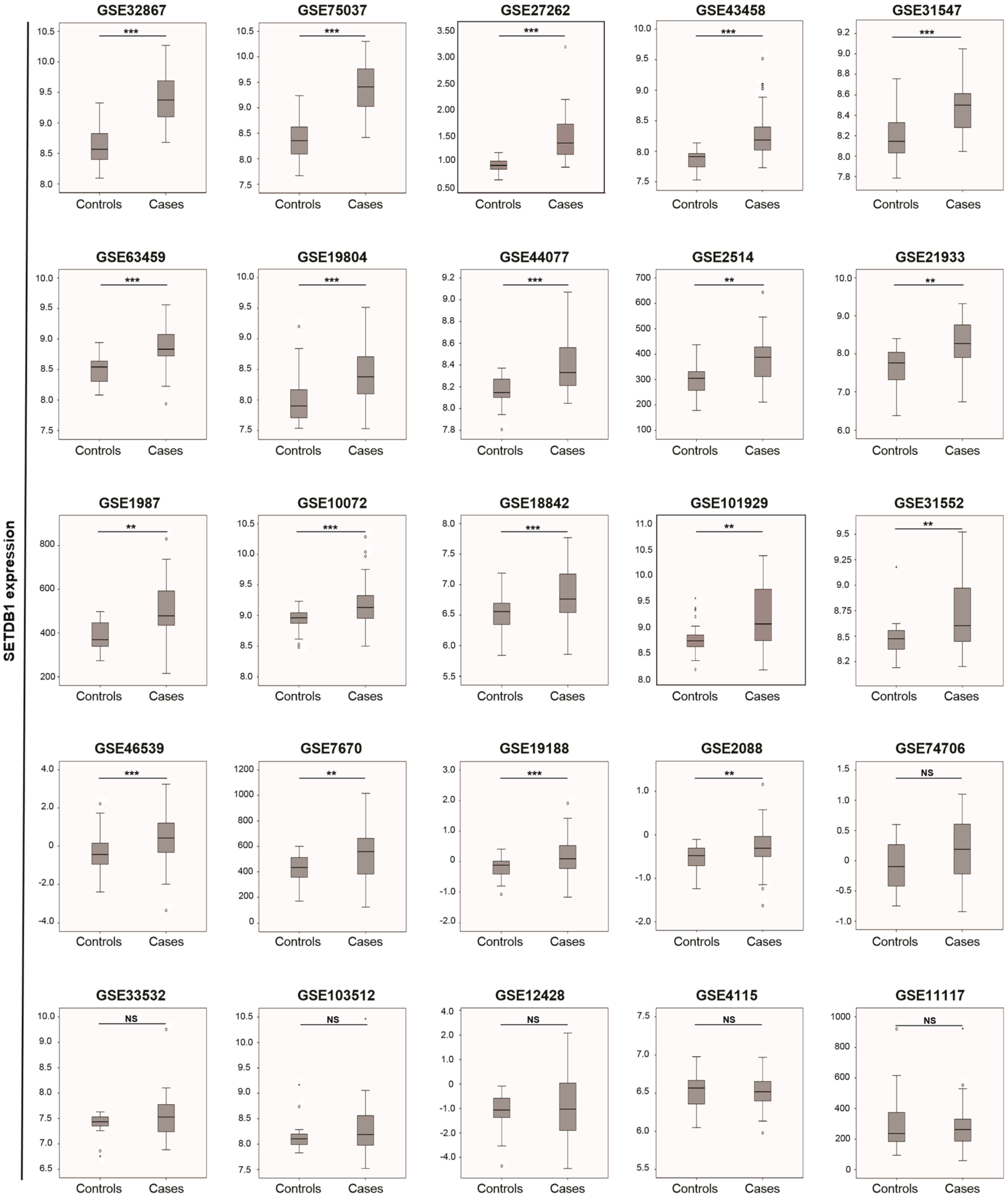

Figure 1. Box plots displaying the expression levels of *SETDB1* mRNA between NSCLC and non-tumor samples for each GEO dataset.

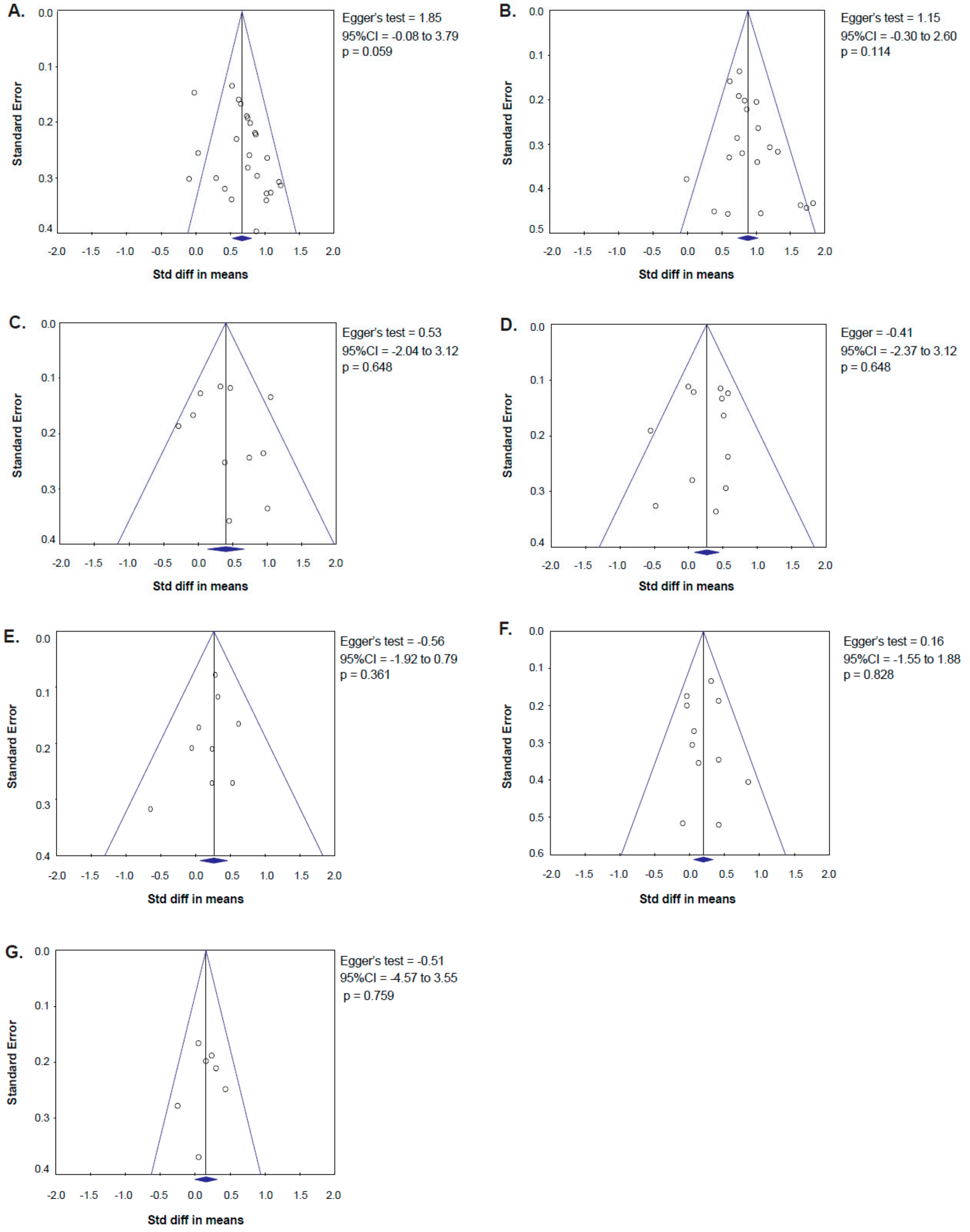

Figure S2. Funnel plots for main meta-analyses performed. (**A**) Association between *SETDB1* mRNA level and NSCLC. (**B**) Association between *SETDB1* mRNA level and ADC. (**C**) Association between SETDB1 mRNA level and SCC. (**D**) Association between *SETDB1* expression in current smokers compared to NSCLC non-smoker patients. (**E**) Association between *SETDB1* mRNA level in former smokers compared to NSCLC non-smoker patients. (**F**) Association between *SETDB1* mRNA level in smokers compared to non-smoker NSCLC patients. (**G**) Association between *SETDB1* mRNA level and NSCLC patients carrying mutations for *TP53* gene.

A.

Study	Std diff in means	Lower limit	Upper limit	p-Value	Weight
GSE31210	0.30	0.04	0.56	0.022	28.35%
GSE68571	0.12	-0.56	0.82	0.719	4.05%
GSE11969	-0.04	-0.38	0.30	0.812	16.70%
GSE8569	0.84	0.04	1.63	0.038	3.09%
GSE101929	-0.09	-1.11	0.91	0.847	1.91%
GSE41271	-0.04	-0.43	0.35	0.839	12.75%
GSE29016	0.41	-0.26	1.09	0.229	4.26%
GSE66863	0.06	-0.46	0.58	0.818	7.05%
GSE23822	0.41	-0.60	1.43	0.424	1.88%
GSE13213	0.41	0.04	0.78	0.027	14.52%
GSE26939	0.03	-0.56	0.63	0.904	5.45%
**Total (95% CI)**	**0.196**	**0.057**	**0.336**	**0.006**	**100%**

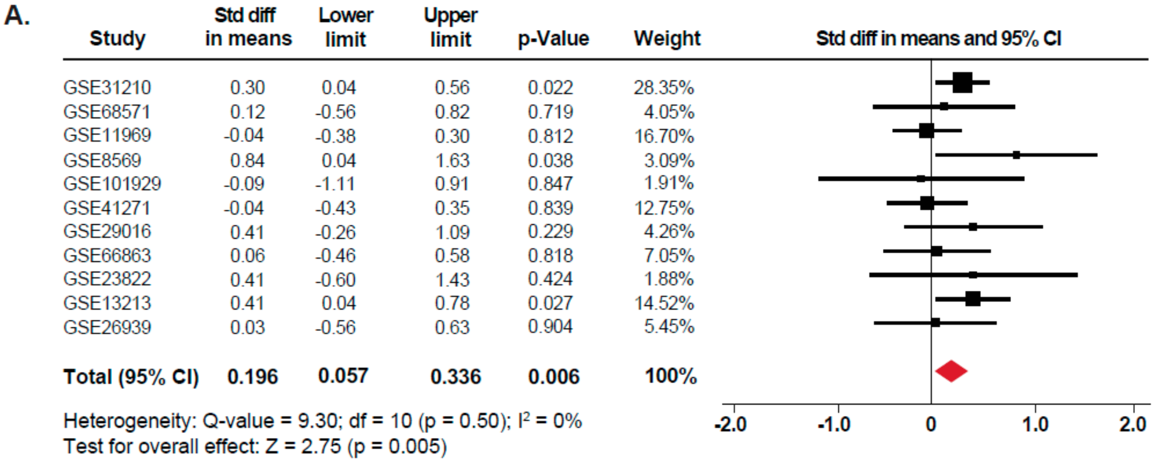

Heterogeneity: Q-value = 9.30; df = 10 (p = 0.50); I^2 = 0%
Test for overall effect: Z = 2.75 (p = 0.005)

B.

Study	Std diff in means	Lower limit	Upper limit	p-Value	Weight
GSE63459	0.05	-0.67	0.77	0.893	4.91%
GSE68571	-0.25	-0.79	0.29	0.368	8.68%
GSE11969	0.04	-0.27	0.37	0.776	24.34%
GSE66863	0.23	-0.13	0.60	0.207	18.97%
GSE13213	0.15	-0.23	0.54	0.433	17.13%
GSE26939	0.30	-0.11	0.71	0.155	15.08%
GSE8569	0.43	-0.05	0.92	0.080	10.89%
**Total (95% CI)**	**0.156**	**-0.004**	**0.317**	**0.056**	**100%**

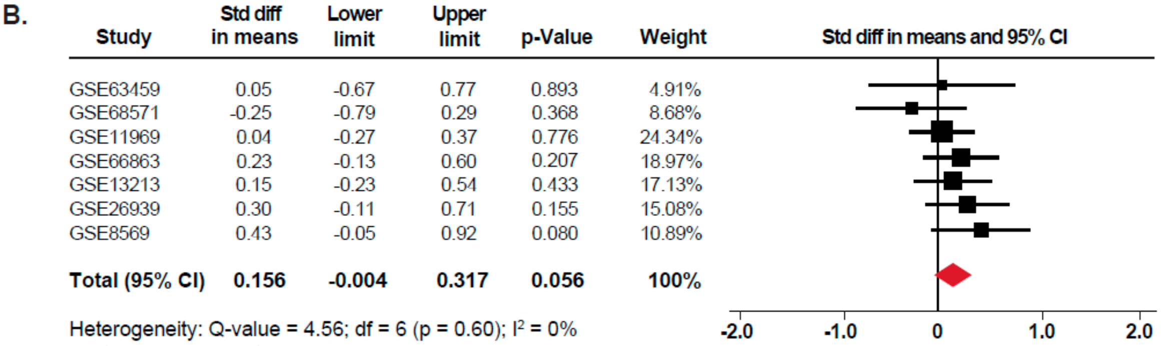

Heterogeneity: Q-value = 4.56; df = 6 (p = 0.60); I^2 = 0%
Test for overall effect: Z = 1.90 (p = 0.056)

Figure S3. (**A**) Forest plot of the association of *SETDB1* mRNA level with NSCLC patients reported as smokers compared to non-smoker patients. (**B**) Forest plot of association between *SETDB1* mRNA level and NSCLC patients carrying mutations for *TP53* gene.

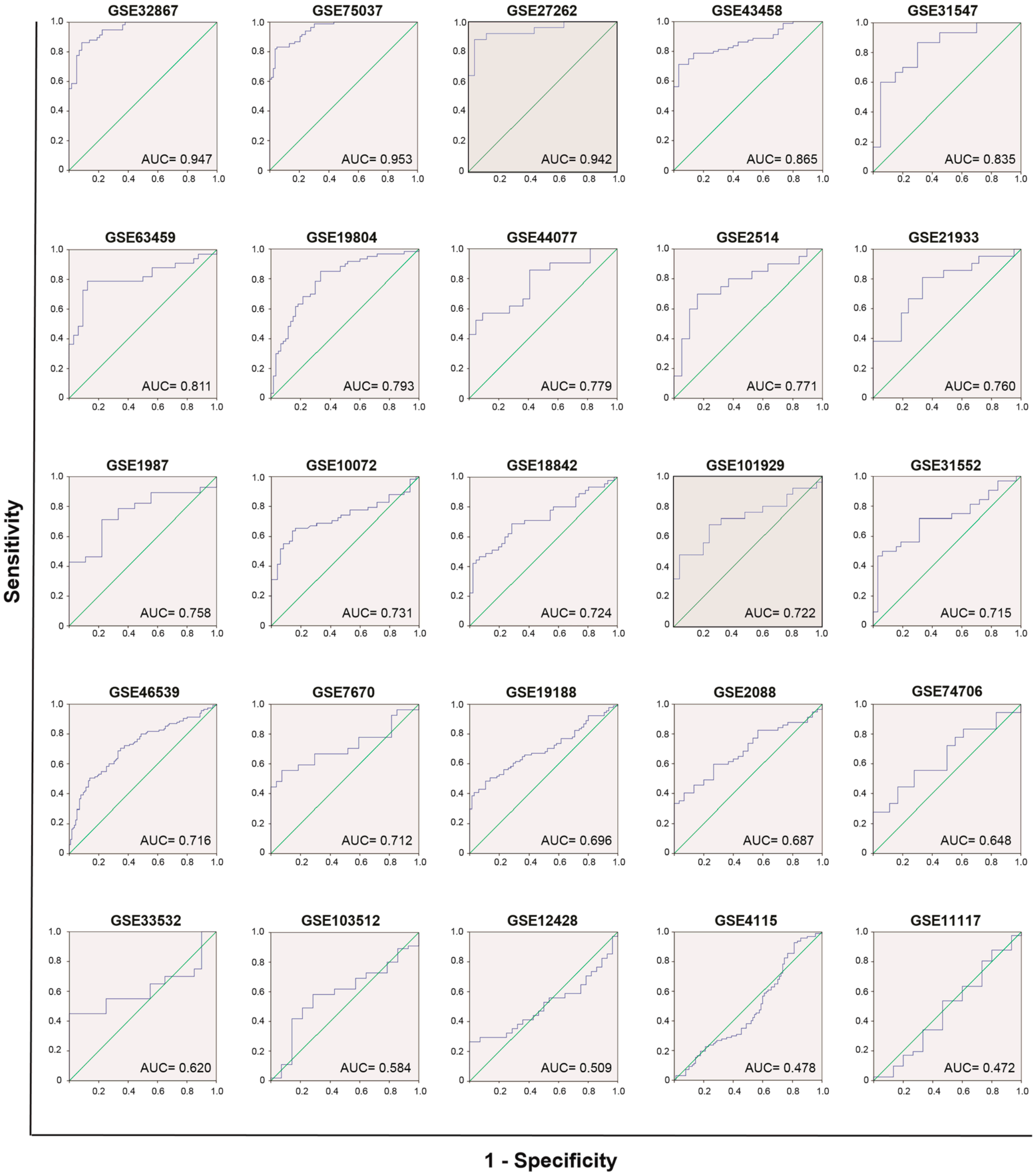

Figure S4. Individual ROC curves showing discriminative yield of *SETDB1* mRNA level for NSCLC for each GEO dataset. AUC: area under the curve.

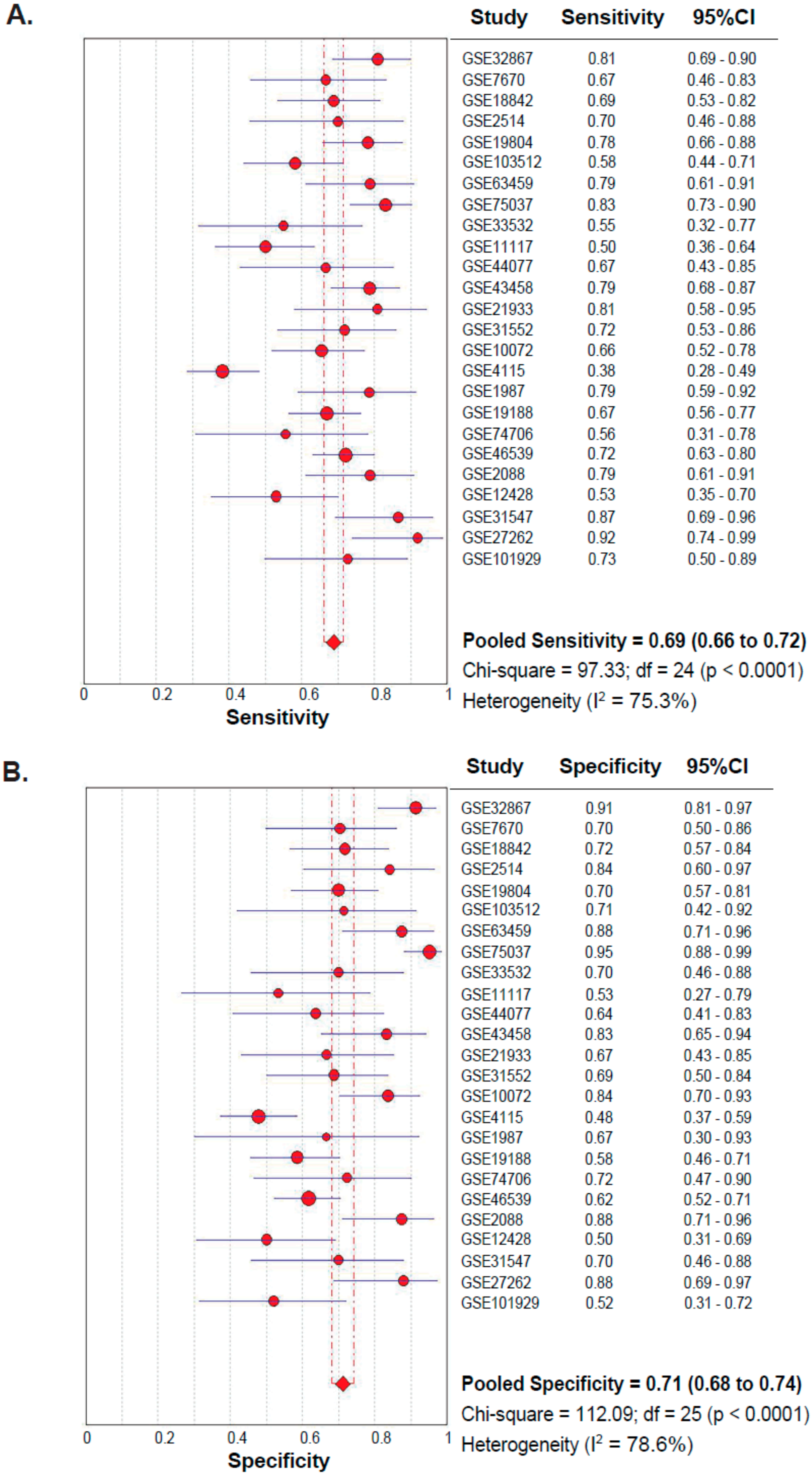

Figure S5. The combined sensitivity and specificity showing moderate discriminative yield of SETDB1 mRNA expression for NSCLC. (**A**) Sensitivity analysis. (**B**) Specificity analysis.

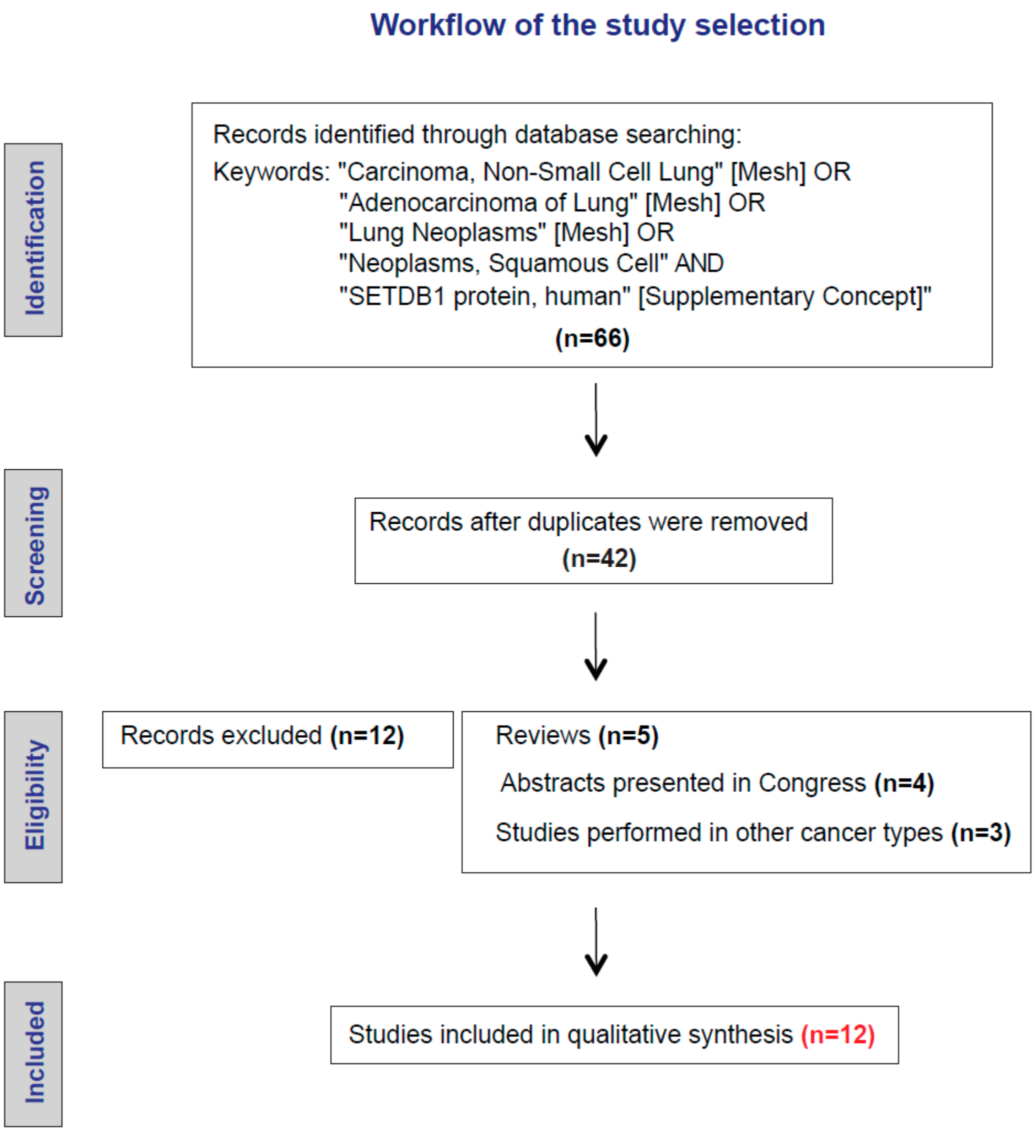

Figure S6. Workflow of the study selection for qualitative synthesis.

Table S1. Overview of additional datasets selected from GEO for clinico-pathological analyses.

GEO Dataset	Country	Year	ADC	SCC	NSCLC	Sample Type Patients	Platform	SETDB1 ID	Reference
GSE31210	Japan	2012	226			Cancer tissue	Affymetrix Human Genome U133 Plus 2.0 Array	203155_at	[1]
GSE68571	USA	2002	86			Cancer tissue	Affymetrix Human Full Length HuGeneFL Array	D31891_at	[2]
GSE11969	Japan	2006	94	35		Cancer tissue	Agilent Homo sapiens 21.6K custom array	A_23_P126398	[3]
GSE8569	Spain	2008	30	36		Cancer tissue	CNIO Human Oncochip 2.0	20543	[4]
GSE47115	USA	2014			46	Cancer tissue	Illumina HumanHT-12 WG-DASL V4.0 R2 expression beadchip	ILMN_1718207	[5]
GSE41271	USA	2013	183	80	1	Cancer tissue	Illumina HumanWG-6 v3.0 expression beadchip	ILMN_1718207	[6–9]
GSE50081	Canada	2014	127	43		Cancer tissue	Affymetrix Human Genome U133 Plus 2.0 Array	203155_at	[10]
GSE68465	USA	2008	443			Cancer tissue	Affymetrix Human Genome U133A Array	203155_at	[11]
GSE29016	Sweden	2012	38	12		Cancer tissue	Illumina HumanHT-12 V3.0 expression beadchip	ILMN_1718207	[12]
GSE66863	Norway	2016	121			Cancer tissue	Agilent-028004 SurePrint G3 Human GE 8x60K Microarray	A_23_P126393	[13]
GSE23822	Australia	2012		56		Cancer tissue	Illumina HumanHT-12 V3.0 expression beadchip	ILMN_1718207	[14]
GSE68793	USA	2015		133		Cancer tissue	Affymetrix HT Human Genome U133A Array	203155_at	Not published
GSE13213	Japan	2009	117			Cancer tissue	Agilent-014850 Whole Human Genome Microarray 4x44K G4112F	A_23_P126393	[15]
GSE26939	USA	2012	116			Cancer tissue	Agilent-UNC-custom-4X44K	A_23_P126393	[16]
GSE14814	Canada	2010	71	52		Cancer tissue	Affymetrix Human Genome U133A Array	203155_at	[17]
GSE37745	Sweden	2017	106	66		Cancer tissue	Affymetrix Human Genome U133 Plus 2.0 Array	203155_at	[18]
GSE115458	Germany	2019	40	30		Cancer tissue	Affymetrix Human Genome U133 Plus 2.0 Array	203155_at	[19]
GSE29013	USA	2011	30	25		Cancer tissue	Affymetrix Human Genome U133 Plus 2.0 Array	203155_at	[20]
GSE67061	China	2015		69		Cancer tissue	Agilent-014850 Whole Human Genome Microarray 4x44K G4112F	A_23_P126393	Not published
GSE5123	Australia	2007		51		Cancer tissue	PRHU05-S1-0006 (PC Human Operon v2 21k)	4.2.19.1	[21]

Table S2. Literature review about SETDB1 status in cell lines and xenograft models.

Study	Study Design, Subjects	Main Findings	Methods and Analysis	Conclusions
**Watanabe *et al.* 2008** [22]	NSCLC cell lines: A549, Calu-1, SK-MES-1, SK-LU-1 and SW-900. Immortalized LT-hT NHBE and transformed Ras-LT-hT NHBE cells	SETDB1 was upregulated in NSCLC cell lines compared to NHBE cells as well as in immortalized and transformed cell lines. SETDB1 regulated cell proliferation in the colony formation assay and apoptosis immortalized and transformed NHBE cell lines	RT-qPCR, ELISA	SETDB1 plays a role in the oncogenic transformation and immortalization of NHBE cells
**Lee *et al.* 2013** [23]	NSCLC cell lines: H1299, H460, A549	SETDB1 involved in the regulation of the apoptosis	MTT, FACS analysis	SETDB1 plays an oncogenic role in lung carcinogenesis
**Wu *et al.* 2014** [24]	Sublines of the CL1 lung cancer cell line (ADC)	SETDB1 protein levels were reduced in the highly metastatic cell lines but increased in the cell lines with low metastatic potential.	WB	SETDB1 plays either pro-oncogenic and anti-oncogenic roles of in different stages of lung cancer
		SETDB1 regulated cellular invasion and the cell metastasis through the direct interaction with SMAD2/3 and regulation of *ANXA2* expression.	in vitro migration/invasion assays, xenograft, luciferase reporter assay, WB, ChIP	
**Rodriguez-Paredes *et al.* 2014** [25]	NSCLC cell lines: A549, NCI-H1299, NCI-H1975, NCI-H1993, NCI-H2170, NCI-H1437 and NCI-H1395. SCLC cell lines: HCC-33, N417, NCI-H446, NCI-H1048, NCI-H1963, NCI-H2029, DMS-114 and DMS-273. Control samples: lung epithelium and leukocytes	SETDB1 gene amplification, mRNA and protein overexpression was found in NCI-H1437, NCI-H1395, DMS-273 cell lines	FISH, qPCR, WB	SETDB1 regulates cell proliferation and cellular invasion in lung cancer cell lines
		Depletion of SETDB1 expression reduces cancer growth in cell culture and nude mice models, whereas its overexpression increases the tumor invasiveness	Colony formation assay, xenograft, matrigel invasion assay	
**Sun *et al.* 2015** [26]	NSCLC cell lines: PC14, A549, HCC2279, H1299, H23 and HCC1975	SETDB1 promoted anchorage-independent growth of NSCLC cell lines and stimulates tumor growth in mice.	Colony formation assay, xenografts	SETDB1 promotes lung carcinogenesis
		There is an inverse correlation between the protein levels of SETDB1 and TP53.	qPCR, WB	
		SETDB1 could promote oncogenesis through activation of the WNT pathway	Microarray, ChIP, WB, luciferase reporter assay	
**Na *et al.* 2018** [27]	A549 NSCLC cell line	ERK2-SETDB1-FosB signaling pathway involved in transformation and migration activity of cancer cells during treatment	ChIP, RT-qPCR, CFA, WB, Scratch and cell migration assays	SETDB1 regulates the chemo-resistance during treatment
**Chen *et al.* 2018** [28]	NSCLC cell lines: A549 and PC14. Bronchial epithelial BEAS-2B cell line	*SETDB1* mRNA and protein levels were elevated in NSCLC cell lines compared to the normal epithelial cell line. SETDB1 promoted oncogenesis *via TP53* and *miR-29s* repression	RT-qPCR, WB, Luciferase reporter assay	SETDB1 plays an oncogenic role in lung carcinogenesis
**Emran *et al.* 2018** [29]	A549 NSCLC cell line	Silencing of SETDB1 reversed multi-drug tolerance.	RT-qPCR, WB	SETDB1 regulates chemo-resistance during treatment
**Wang *et al.* 2019** [30]	A549 NSCLC cell line	SETDB1 promoted oncogenesis by methylation of Akt K64 and the Akt hyperactivation	Colony formation assay, IP, WB, methylation assay	SETDB1 accelerates oncogenesis
**Noh *et al.* 2014** [31]	A549 NSCLC cell line	Paclitaxel induced down-regulation of SETDB1 expression at transcriptional and protein levels	RT-qPCR and WB	Potential of SETDB1 as target for anti-cancer drugs

References

1. Okayama, H.; Kohno, T.; Ishii, Y.; Shimada, Y.; Shiraishi, K.; Iwakawa, R.; Furuta, K.; Tsuta, K.; Shibata, T.; Yamamoto, S.; et al. Identification of genes upregulated in ALK-positive and EGFR/KRAS/ALK-negative lung adenocarcinomas. *Cancer Res*. **2012**, *72*, 100–111.
2. Beer, D.G.; Kardia, S.L.R.; Huang, C.-C.; Giordano, T.J.; Levin, A.M.; Misek, D.E.; Lin, L.; Chen, G.; Gharib, T.G.; Thomas, D.G.; et al. Gene-expression profiles predict survival of patients with lung adenocarcinoma. *Nat. Med.* **2002**, *8*, 816–824.
3. Takeuchi, T.; Tomida, S.; Yatabe, Y.; Kosaka, T.; Osada, H.; Yanagisawa, K.; Mitsudomi, T.; Takahashi, T. Expression profile-defined classification of lung adenocarcinoma shows close relationship with underlying major genetic changes and clinicopathologic behaviors. *J. Clin. Oncol*. **2006**, *24*, 1679–1688.
4. Angulo, B.; Suarez-Gauthier, A.; Lopez-Rios, F.; Medina, P.P.; Conde, E.; Tang, M.; Soler, G.; Lopez-Encuentra, A.; Cigudosa, J.C.; Sanchez-Cespedes, M.; et al. Expression signatures in lung cancer reveal a profile for EGFR-mutant tumours and identify selective PIK3CA overexpression by gene amplification. *J. Pathol*. **2008**, *21*, 4347–4356.
5. Yu, G.; Herazo-Maya, J.D.; Nukui, T.; Romkes, M.; Parwani, A.; Juan-Guardela, B.M.; Robertson, J.; Gauldie, J.; Siegfried, J.M.; Kaminski, N.; et al. Matrix metalloproteinase-19 promotes metastatic behavior in vitro and is associated with increased mortality in non-small cell lung cancer. *Am. J. Respir. Crit. Care Med*. **2014**, *190*, 780–790.
6. Sato, M.; Larsen, J.E.; Lee, W.; Sun, H.; Shames, D.S.; Dalvi, M.P.; Ramirez, R.D.; Tang, H.; DiMaio, J.M.; Gao, B.; et al. Human lung epithelial cells progressed to malignancy through specific oncogenic manipulations. *Mol. Cancer Res.* **2013**, *11*, 638–650.
7. Riquelme, E.; Suraokar, M.; Behrens, C.; Lin, H.Y.; Girard, L.; Nilsson, M.B.; Simon, G.; Wang, J.; Coombes, K.R.; Lee, J.J.; et al. VEGF/VEGFR-2 upregulates EZH2 expression in lung adenocarcinoma cells and EZH2 depletion enhances the response to platinum-based and VEGFR-2-targeted therapy. *Clin. Cancer Res.* **2014**, *20*, 3849–3861.
8. Girard, L.; Rodriguez-Canales, J.; Behrens, C.; Thompson, D.M.; Botros, I.W.; Tang, H.; Xie, Y.; Rekhtman, N.; Travis, W.D.; Wistuba, I.I.; et al. An Expression Signature as an Aid to the Histologic Classification of Non-Small Cell Lung Cancer. *Clin. Cancer Res.* **2016**, *22*, 4880–4889.
9. Parra, E.R.; Behrens, C.; Rodriguez-Canales, J.; Lin, H.; Mino, B.; Blando, J.; Zhang, J.; Gibbons, D.L.; Heymach, J.V.; Sepesi, B.; et al. Image Analysis-based Assessment of PD-L1 and Tumor-Associated Immune Cells Density Supports Distinct Intratumoral Microenvironment Groups in Non-small Cell Lung Carcinoma Patients. *Clin. Cancer Res.* **2016**, *22*, 6278–6289.
10. Der, S.D.; Sykes, J.; Pintilie, M.; Zhu, C.-Q.; Strumpf, D.; Liu, N.; Jurisica, I.; Shepherd, F.A., Tsao, M.S. Validation of a histology-independent prognostic gene signature for early-stage, non-small-cell lung cancer including stage IA patients. *J. Thorac. Oncol.* **2014**, *9*, 59–64.
11. Director's Challenge Consortium for the Molecular Classification of Lung Adenocarcinoma; Shedden, K.; Taylor, J.M.G.; Enkemann, S.A.; Tsao, M.-S.; Yeatman, T.J.; Gerald, W.L.; Eschrich, S.; Jurisica, I.; Giordano, T.J.; et al. Gene expression-based survival prediction in lung adenocarcinoma: A multi-site, blinded validation study. *Nat. Med.* **2008**, *14*, 822–827.
12. Staaf, J.; Jönsson, G.; Jönsson, M.; Karlsson, A.; Isaksson, S.; Salomonsson, A.; Pettersson, H.M.; Soller, M.; Ewers, S.B.; Johansson, L.; et al. Relation between smoking history and gene expression profiles in lung adenocarcinomas. *BMC Med. Genomics* **2012**, *5*, 22.
13. Bjaanæs, M.M.; Fleischer, T.; Halvorsen, A.R.; Daunay, A.; Busato, F.; Solberg, S.; Jørgensen, L.; Kure, E.; Edvardsen, H.; Børresen-Dale, A.L.; et al. Genome-wide DNA methylation analyses in lung adenocarcinomas: Association with EGFR, KRAS and TP53 mutation status, gene expression and prognosis. *Mol. Oncol.* **2016**, *10*, 330–343.
14. Wright, C.M.; Savarimuthu Francis, S.M.; Tan, M.E.; Martins, M.U.; Winterford, C.; Davidson, M.R.; Duhig, E.E.; Clarke, B.E.; Hayward, N.K.; Yang, I.A.; et al. MS4A1 dysregulation in asbestos-related lung squamous cell carcinoma is due to CD20 stromal lymphocyte expression. *PLoS ONE* **2012**, *7*, e34943, doi:10.1371/journal.pone.0034943.
15. Tomida, S.; Takeuchi, T.; Shimada, Y.; Arima, C.; Matsuo, K.; Mitsudomi, T.; Yatabe, Y.; Takahashi, T. Relapse-related molecular signature in lung adenocarcinomas identifies patients with dismal prognosis. *J. Clin. Oncol.* **2009**, *27*, 2793–2799.
16. Wilkerson, M.D., Yin, X.; Walter, V.; Zhao, N.; Cabanski, C.R.; Hayward, M.C.; Miller, C.R.; Socinski, M.A.;

Parsons, A.M.; Thorne, L.B.; et al. Differential pathogenesis of lung adenocarcinoma subtypes involving sequence mutations, copy number, chromosomal instability, and methylation. *PLoS ONE* **2012**, *7*, e36530, doi:10.1371/journal.pone.0036530.
17. Zhu, C.-Q.; Ding, K.; Strumpf, D.; Weir, B.A.; Meyerson, M.; Pennell, N.; Thomas, R.K.; Naoki, K.; Ladd-Acosta, C.; Liu, N.; et al. Prognostic and predictive gene signature for adjuvant chemotherapy in resected non-small-cell lung cancer. *J. Clin. Oncol.* **2010**, *28*, 4417–4424.
18. Botling, J.; Edlund, K.; Lohr, M.; Hellwig, B.; Holmberg, L.; Lambe, M.; Berglund, A.; Ekman, S.; Bergqvist, M.; Pontén, F.; et al. Biomarker discovery in non-small cell lung cancer: Integrating gene expression profiling, meta-analysis, and tissue microarray validation. *Clin. Cancer Res.* **2013**, *19*, 194–204.
19. Weber, R.; Meister, M.; Muley, T.; Thomas, M.; Sültmann, H.; Warth, A.; Winter, H.; Herth, F.J.F.; Schneider, M.A. Pathways regulating the expression of the immunomodulatory protein glycodelin in non-small cell lung cancer. *Int. J. Oncol.* **2019**, *54*, 515–526.
20. Xie, Y.; Xiao, G.; Coombes, K.R.; Behrens, C.; Solis, L.M.; Raso, G.; Girard, L.; Erickson, H.S.; Roth, J.; Heymach, J.V.; et al. Robust gene expression signature from formalin-fixed paraffin-embedded samples predicts prognosis of non-small-cell lung cancer patients. *Clin. Cancer Res.* **2011**, *17*, 5705–5714.
21. Larsen, J.E.; Pavey, S.J.; Passmore, L.H.; Bowman, R.; Clarke, B.E.; Hayward, N.K.; Fong, K.M. Expression profiling defines a recurrence signature in lung squamous cell carcinoma. *Carcinogenesis* **2007**, *28*, 760–766.
22. Watanabe, H.; Soejima, K.; Yasuda, H.; Kawada, I.; Nakachi, I.; Yoda, S.; Naoki, K.; Ishizaka, A. Deregulation of histone lysine methyltransferases contributes to oncogenic transformation of human bronchoepithelial cells. *Cancer Cell Int.* **2008**, *8*, 15.
23. Lee, J.-K.; Kim, K.-C. DZNep, inhibitor of S-adenosylhomocysteine hydrolase, down-regulates expression of SETDB1 H3K9me3 HMTase in human lung cancer cells. *Biochem. Biophys. Res. Commun.* **2013**, *438*, 647–652.
24. Wu, P.C.; Lu, J.W.; Yang, J.Y.; Lin, I.H.; Ou, D.L.; Lin, Y.H.; Chou, K.H.; Huang, W.F.; Wang, W.P.; Huang, Y.L.; et al. H3K9 histone methyltransferase, KMT1E/SETDB1, cooperates with the SMAD2/3 pathway to suppress lung cancer metastasis. *Cancer Res.* **2014**, *74*, 7333–7343.
25. Rodriguez-Paredes, M.; Martinez de Paz, A.; Simó-Riudalbas, L.; Sayols, S.; Moutinho, C.; Moran, S.; Villanueva, A.; Vázquez-Cedeira, M.; Lazo, P.A.; Carneiro, F.; et al. Gene amplification of the histone methyltransferase SETDB1 contributes to human lung tumorigenesis. *Oncogene* **2014**, *33*, 2807–2813.
26. Sun, Q.Y.; Ding, L.W., Xiao, J.F.; Chien, W.; Lim, S.L.; Hattori, N.; Goodglick, L.; Chia, D.; Mah, V.; Alavi, M.; et al. SETDB1 accelerates tumourigenesis by regulating the WNT signalling pathway. *J. Pathol.* **2015**, *235*, 559–570.
27. Na, H.H.; Kim, K.C. SETDB1-mediated FosB regulation via ERK2 is associated with an increase in cell invasiveness during anticancer drug treatment of A549 human lung cancer cells. *Biochem Biophys Res. Commun.* **2018**, *495*, 512–518.
28. Chen, B.; Wang, J.; Wang, J.; Wang, H.; Gu, X.; Tang, L.; Feng, X. A regulatory circuitry comprising TP53, miR-29 family, and SETDB1 in non-small cell lung cancer. *Biosci. Rep.* **2018**, *38*, doi:10.1042/BSR20180678.
29. Al Emran, A.; Marzese, D.M.; Menon, D.R.; Stark, M.S.; Torrano, J.; Hammerlindl, H.; Zhang, G.; Brafford, P.; Salomon, M.P.; Nelson, N.; et al. Distinct histone modifications denote early stress-induced drug tolerance in cancer. *Oncotarget* **2018**, *9*, 8206–8222.
30. Wang, G.; Long, J.; Gao, Y.; Zhang, W.; Han, F.; Xu, C.; Sun, L.; Yang, S.C.; Lan, J.; Hou, Z.; et al. SETDB1-mediated methylation of Akt promotes its K63-linked ubiquitination and activation leading to tumorigenesis. *Nat. Cell Biol.* **2019**, *21*, 214–225.
31. Noh, H.-J., Kim K-A, Kim K-C. p53 down-regulates SETDB1 gene expression during paclitaxel induced-cell death. *Biochem. Biophys. Res. Commun.* **2014**, *446*, 43–48.

© 2019 by the authors. Licensee MDPI, Basel, Switzerland. This article is an open access article distributed under the terms and conditions of the Creative Commons Attribution (CC BY) license (http://creativecommons.org/licenses/by/4.0/).
